# VisANT 4.0: Integrative network platform to connect genes, drugs, diseases and therapies

**DOI:** 10.1093/nar/gkt401

**Published:** 2013-05-28

**Authors:** Zhenjun Hu, Yi-Chien Chang, Yan Wang, Chia-Ling Huang, Yang Liu, Feng Tian, Brian Granger, Charles DeLisi

**Affiliations:** Center for Advanced Genomic Technology, Bioinformatics Program, Boston University, Boston, MA 02215, USA

## Abstract

With the rapid accumulation of our knowledge on diseases, disease-related genes and drug targets, network-based analysis plays an increasingly important role in systems biology, systems pharmacology and translational science. The new release of VisANT aims to provide new functions to facilitate the convenient network analysis of diseases, therapies, genes and drugs. With improved understanding of the mechanisms of complex diseases and drug actions through network analysis, novel drug methods (e.g., drug repositioning, multi-target drug and combination therapy) can be designed. More specifically, the new update includes (i) integrated search and navigation of disease and drug hierarchies; (ii) integrated disease–gene, therapy–drug and drug–target association to aid the network construction and filtering; (iii) annotation of genes/drugs using disease/therapy information; (iv) prediction of associated diseases/therapies for a given set of genes/drugs using enrichment analysis; (v) network transformation to support construction of versatile network of drugs, genes, diseases and therapies; (vi) enhanced user interface using docking windows to allow easy customization of node and edge properties with build-in legend node to distinguish different node type. VisANT is freely available at: http://visant.bu.edu.

## INTRODUCTION

There is increasing evidence that most diseases result from abnormalities of many genes rather than a single gene. These genes work collaboratively, as a complicated network, to reflect the functional variation of corresponding cellular processes (1,2). Such network-based views and approaches enhance our capability to identify disease-associated genes, modules and pathways; they also bring new insights of the functional and causal relationship among different diseases because of the interdependency between a cell’s molecular components (3).

Network-based approaches also lead to the relatively new emerging field, network pharmacology, where drug actions and side effects have been analysed in the context of regulatory networks, within which drug targets and disease genes function through complicated inter-connection (4). Such approaches provide new tools for the drug discovery of complex diseases, and the study of the efficacy of drugs that may have more than one binding partners. A number of new ideas, such as drug repositioning (5–9), multi-target drugs (10–12) and combination therapy (i.e., the use of several drugs together to treat a single disease) (13) are therefore becoming promising areas of drug design.

**Figure 1.**
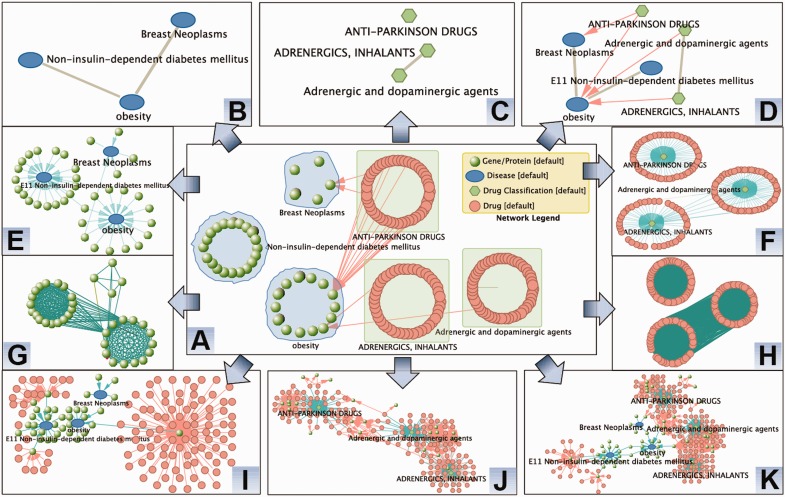
Illustration of versatile network construction in VisANT 4.0 with integrated disease and therapy hierarchies, disease–gene and therapy–drug associations and drug–target interactions. Detailed explanation can be found in the session ‘Network construction’. (**A**) Meta-network of diseases and therapies. Expanded metanodes of diseases are represented using convex polygons. Drugs are queried for their targets in three diseases (red lines). The rest of the networks (B–K) are all derived from this meta-network. (**B**) Disease network where grey edges indicate that there is shared genes associated with two diseases. (**C**) Therapy network similar to disease network. (**D**) Disease–therapy network. (**E**) Disease–gene network. (**F**) Therapy–drug network. (**G**) Co-disease gene network. (**H**) Co-therapy drug network. (**I**) Disease–gene–drug network. (**J**) Therapy–drug–gene network. (**K**) Disease–gene–drug–therapy network.

Although tools designed for network analysis have been blooming since early 2004 as a response to the emerging popularity of the interaction data of biomolecules (e.g., protein–protein interaction) (14–16), none of them provide the integrated knowledge and functionality to support systematic analysis of the versatile type of networks of diseases, therapies, genes and drugs (as shown in Figure 1) that have a great promise to aid the understanding of the mechanism of disease complexity and drug action, and to discover novel drug targets and improve the approval rate of new drugs (1,17). The great challenge is to integrate the hierarchical knowledge (e.g., disease hierarchy and therapy hierarchy) with adjacency relations (e.g., protein–protein interaction and drug–target interaction), which requires the use of advanced graph types as detailed in our previous publication (15).

Here, we report VisANT’s metagraph-based solution with integrated disease and therapy hierarchy, disease–gene and therapy–drug association that allows the convenient construction of 11 different types of networks to analyse the correlations between disease, therapy, genes and drugs systematically (Figure 1). A list of new functions have been implemented to assist the versatile network analyses to improve our understanding of the mechanisms of complex diseases and drug actions, including (i) integrated search and navigation of disease and drug hierarchies; (ii) integrated disease–gene, therapy–drug and drug–target association to aid the network construction and filtering; (iii) annotation of genes/drugs using disease/therapy information; (iv) prediction of associated diseases/therapies for a given set of genes/drugs using enrichment analysis; (v) network transformation to support construction of various network of drugs, genes, diseases and therapies; (vi) enhanced user interface using docking windows to allow easy customization of node and edge properties with build-in legend node to distinguish different node type.

VisANT is a Web-based workbench for the integrative analysis of biological networks with several unique features, and this update provides it with new capabilities of translational sciences to convert our understanding of basic biological knowledge into effective ways to treat and prevent diseases. The first version of VisANT was released in early 2004 and supported exploratory navigation to walk through the interactions based on a few initial genes or proteins of interest (18); the second release implemented a primary version of metagraph, as we realized the importance to integrate the context information (such as protein complex, functional modules etc.) into the network (19). This release also featured the unique topological analyses (e.g., exhaustive search of shortest paths between two nodes) that is dynamically linked to the network; the importance of the metagraph was quickly recognized by the community (15), and in VisANT release 3 (20) and 3.5 (21), it was successfully applied to achieve intuitive visualization of KEGG pathway and multi-scale network visual analysis and inference with integrated Gene Ontology (GO). In VisANT 3.5, we also implemented a sophisticated GO explorer to facilitate the visual navigation and application of GO hierarchy, which is renamed as Hierarchy Explorer in this update to reflect the addition of disease and therapy hierarchies.

## PROGRAM DESCRIPTION

### Data sources

The disease hierarchy is represented by ICD-10 (International Classification of Disease), and therapy hierarchy is represented by ATC (Anatomical Therapeutic Chemical) classification. Both classifications are specified by the WHO (World Health Organization) and were downloaded from its website. Disease–gene associations were gathered from KEGG Disease database (22), OMIM (Online Mendelian Inheritance in Man) (23), PharmGKB (24) and GAD (Genetic Association Database) (25), while therapy–drug and drug–target associations were collected from KEGG Drug database (22) and DrugBank database (26). In addition, html links of the original sources are always available for corresponding nodes and edges. Standard data integration and synchronization procedures (27) are carried out for diseases, drugs, therapies and their association data to keep the information in VisANT system up to date.

### Visual navigation of disease and therapy hierarchy

**Figure 2.**
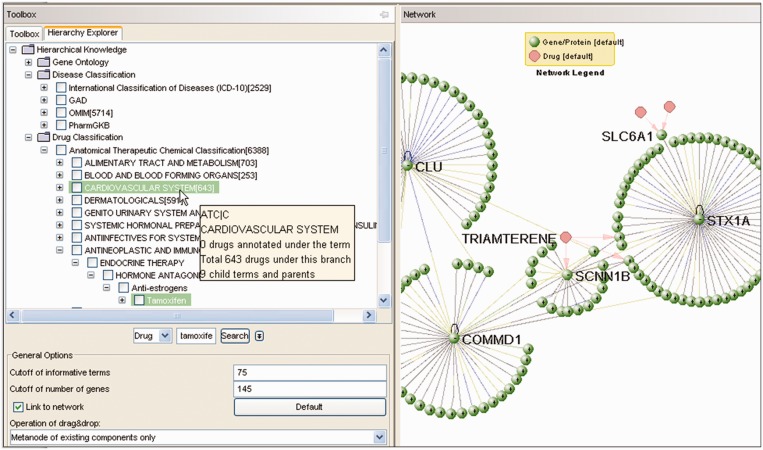
Hierarchy Explorer and exploratory navigation in VisANT. Three types of hierarchies are available: GO, disease and drugs. The example of the exploratory navigation follows the order: STX1A**→**SLC6A1**→**SCNN1B**→**TRIAMTERENE**→**COMMD1**→**CLU.

The Hierarchy Explorer uses the tree structure to visualize and navigate the hierarchy of GO, disease and therapy (Figure 2). A tab control is introduced to switch between the Hierarchy Explorer and the Toolbox of the VisANT in the left control panel in VisANT. The width of the panel can be changed through mouse dragging to facilitate easy browsing while the width of the toolbox will remain unchanged. Clicking on the expansion symbol or double clicking over the tree node will expand/collapse it. A database query will be sent to the VisANT server to retrieve its entire child terms when the tree node is expanded the first time, and a number will be shown in each node to indicate the number of genes annotated under the branch for the current species. When a different species is selected, the number will be changed accordingly. Other information, such as the number of components (genes or drugs) directly annotated under the term, is shown in the tooltip when the user mouse-overs the tree node (Figure 2).

The queried hierarchy tree is loaded from the VisANT server, so that whenever VisANT starts, it will always get the latest hierarchy knowledge available. Hierarchy information stored in the Predictome database (28) is being updated monthly to match up with the latest modification of the corresponding database. The search box at the bottom of the Hierarchy Explorer allows the user to search the hierarchy tree using key words or term IDs (such as GO IDs). Searching with the gene name, however, is not supported in this search box, but instead can be achieved through the one in VisANT toolbox. On the other hand, drug names may be queried in the Hierarchy Explorer because many drug names overlap with the ATC terms [(e.g., Tamoxifen) as shown in Figure 2].

When a gene is annotated with either GO terms or associated diseases, usually only the most detailed information will be used. However, on some occasions, this annotation is not informative because knowledge hierarchy is discarded. From this perspective, VisANT has been designed to display all possible paths from the corresponding annotations to the root of corresponding hierarchies in the Hierarchy explorer when the user clicks a drug/gene node with annotations, or a metanode of GO term/disease/therapy, or a metanode with results from annotation enrichment analysis, and the option is chosen to link to network (Figure 2). Therefore, we not only know that Tamoxifen is an anti-estrogens drug, but that it is also part of endocrine therapy. The paths are highlighted in the tree to allow users to view and explore the areas of the hierarchy graph surrounding the terms. The number of genes/drugs annotated under terms in the expanded paths is not shown to distinguish these paths from normal term expansion. The users can collapse and expand those terms to get this information. This function indirectly allows the user to search the hierarchical terms using the drug or gene and its products.

Standardization is one of the key issues in the integration of biomedical knowledge. ICD-10 and ATC are the latest standard for disease and drug classification provided by the WHO. However, only a limited number of disease databases support the ICD-10 classification. As a temporary solution, four disease classifications are listed corresponding to the four databases where disease–gene associations are collected (KEGG Disease database adopts the IDC-10). We will merge these disease hierarchies under ICD-10 once they adopt the ICD-10 classification for the diseases (e.g., OMIM will soon adopt the ICD-10 according to its website).

### Exploratory navigation

Starting from an initial gene/drug of interest, exploratory navigation allows you to walk through the interactions one by one. The interactions between genes are either physical or genetic, and the interactions between genes and drugs indicate the corresponding binding targets of the drugs. An example has been shown in Figure 2 following the order STX1A**→** SLC6A1**→** SCNN1B**→** TRIAMTERENE**→** COMMD1**→** CLU.

### Network construction

**Figure 3.**
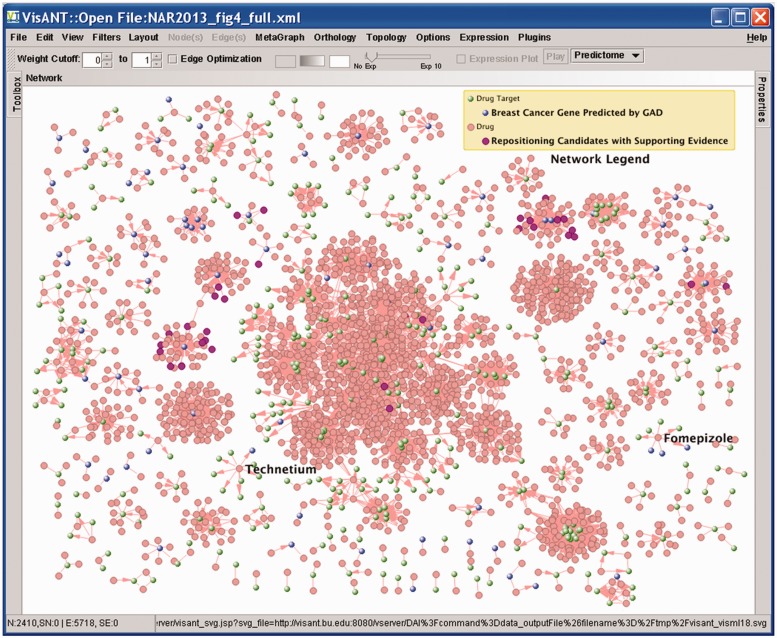
A drug–target network with increased work space in VisANT.

Choice of network representation is often dictated by the research problem at hand. A disease–disease network will help to determine whether they share a common genetic origin while a disease–gene network may tell the common genes shared by them (3,29). On the other hand, a drug–target network may be preferred for the purpose of drug repositioning detailed in our case study (Figure 3). This global drug–target network also reveals some clear characteristics of the drug design similar to what has been found by the work of Yildirim *et al*. (30): the majority of the drugs target only a few proteins, indicating most of drugs are ‘follow-on’ drugs because they target already known targets. There are also a few of drugs, noticeably Technetium and Fomepizole in Figure 3, that target many proteins. Many more networks may be constructed to fit various research purpose, as discussed by Spiro *et al*. (17) and Barabasi *et al*. (1)

With the full advantage of its metagraph implementation and two new network transformation functions, VisANT 4.0 supports the convenient construction of 10 network types in addition to the meta-network as shown in Figure 1 and detailed below:

- Meta-network, where diseases and therapies are represented as metanodes with embedded genes/drugs (Figure 1A). The nodes for diseases/therapies can easily be created through drag&drop operations from the Hierarchy Explorer to the network panel in VisANT. There are four total options for the drag&drop operation, and two of them allows for the embedding of sub-terms in the metanodes to achieve the multi-scale visualization (21). The meta-network serves as the basis for the construction of the rest of the network types.
- Disease–disease network, where only collapsed metanodes of diseases are retained (Figure 1B). The correlations between diseases are inferred based on shared components (genes) between two metanodes, or based on the interactions between components in two metanodes (3,15).
- Therapy–therapy network, where only collapsed metanodes of therapies are retained (Figure 1C). The correlations between therapies are inferred based on shared components (drugs) between two metanodes.
- Disease–therapy network, where both collapsed metanodes of therapies and diseases are retained (Figure 1D). The correlations between therapies are inferred based on integrated drug–target associations.
- Disease–gene network, where metanodes of diseases are transformed through the menu ***MetaGraph→ Network Transformation→ Create Bipartite Graph***, and metanodes of therapies are discarded (Figure 1E). An edge between a gene and a disease indicate that the gene is known to be associated with the disease.
- Therapy–drug network, where metanodes of therapies are transformed through the menu ***MetaGraph→ Network Transformation→ Create Bipartite Graph***, and metanodes of diseases are discarded (Figure 1F). An edge between a drug and a therapy indicates that the drug is known to be part of the therapy.
- Co-disease gene network, where metanodes of diseases are transformed through the menu ***MetaGraph→ Network Transformation→ Create Co-Metanode Network***, and metanodes of therapies are discarded (Figure 1G). An edge between two genes indicates that both are known to be associated with the same disease.
- Co-therapy drug network, where metanodes of therapies are transformed through the menu ***MetaGraph→ Network Transformation→ Create Co-Metanode Network***, and metanodes of diseases are discarded (Figure 1H). An edge between two drugs indicates that both are known to be associated with the same therapy.
- Disease–gene–drug network, where genes in the disease–gene network are queried for the potential drugs targeting them (Figure 1I).
- Therapy–drug–gene network, where drugs in the therapy–drug network are queried for their targets (Figure 1J).
- Disease–gene–drug–therapy network, where metanodes of both diseases and therapies are transformed through the menu ***MetaGraph→ Network Transformation→ Create Bipartite Graph***. Drugs are queried for their targets, and genes are queried for the drugs (Figure 1K).

### Enhanced network filtering and analysis

Edges in the VisANT network can be easily filtered based on the methods associated with them (through the menu ***View→ Method Table***) or based on their weight if it has been assigned (19–21,27). This update therefore focused on the development of the functions to facilitate the node filtering. There are two ways to achieve this.

The first approach is to filter the nodes based on their annotation: GO annotations and disease classifications for genes/proteins, and therapy classifications for drugs. For a given network, this filtering is carried out through the drag&drop operation with the option ‘***Metanode of existing components only***’ (Figure 2). As an example, the blue nodes shown in Figure 3 are the drug targets associated with breast cancer as predicted by the GAD database, resulting from the drag&drop of the term ‘breast cancer’ in the GAD hierarchy to the drug–target network. Detailed procedures of this filtering can be found in the supporting materials. There are also menus to allow users to select genes/proteins that have no disease/GO annotations, or drugs that have no ATC classifications.

The second approach is to filter the nodes based on the data type (e.g., protein/gene, drug, disease etc.) or visual customization (e.g., colour, size, shape etc.). Two menus have been added (***Nodes→Select/Deselect Nodes of The Same Properties***) to allow the user to select/deselect nodes with the same data or visual properties based on the current node selection. If the current selected node has a particular visual customization (node shape, size, and colour), the menu will select/deselect other nodes with the same data type and visual customization.

It is important for many topological analyses to be performed on a specific type of node in a network with multiple node types. From this perspective, the functions of these analyses have been enhanced to allow them to be applied on a select subset of the nodes. In this way, we will be able to, for example, easily find out which drugs have most targets and which genes are being targeted by the most drugs through the degree distribution in the drug–target network shown in Figure 3 (19).

### Network annotation and enrichment analysis

Functions have been provided to add or remove GO or disease annotations to the gene/protein nodes, or ATC classifications for the drug nodes. For any metanodes, hypergeometric test-based annotation enrichment analysis can be performed to predict the associated functions/diseases for a given set of genes, or therapies for a given set of drugs. The details of the algorithm can be found in our previous publication (21), and the instruction for the analysis can be found in VisANT’s user manual.

### GUI (Graphic User Interface) enhancement

VisANT 4.0 adopts a system of docking windows for all major GUI components, which brings two advantages: (i) increase the work space for the network analysis. As shown in Figure 3, both toolbox and property customization windows can be hidden in the left and right side bar, respectively, to provide the maximum work space for the network; (ii) Allow easy customizations of node and edge properties with a significantly reduced number of mouse clicks. The hidden window for the property customization can be easily activated by mouse over/clicking on the button at the right side bar. The categorized property table makes customization more user friendly.

It is necessary to distinguish the types of nodes in a network composed of mixed data types and various visual customizations, such as the drug–target network shown in Figure 3. VisANT 4.0 will automatically create the network legend node such as the one shown in both Figures 2 and 3 using the menu ***View→Network Legend***. The legend node is a metanode and can therefore be collapsed/expanded. The appearance of the legend node, as well as its embedded node, can also be customized like any general node.

## CASE STUDY

In this case study, we will try to find the repositioning drug candidates for breast cancer with the assumption that drugs whose targets are associated with breast cancer may be applied to treat the disease. We first build the drug–target network by loading all drug–target interactions in VisANT through the Method Table (using menu ***View→Method Table***). We then filtered the network to find the genes predicted to be associated with breast cancer by the GAD database (blue nodes in Figure 3). The drugs binding to the blue nodes will be our repositioning candidates. A quick comparison against the results from an independent repositioning study (5) indicates a total 32 overlaps of repositioning candidates with supporting evidence [represented by purple drug nodes in Figure 3, detail of the supporting evidence can be found in (5)]. The step-by-step instruction for this case study can be found in the Supplementary Materials.

## CONCLUSION

The new release of VisANT introduces extensive functionalities to facilitate the construction, visualization and analysis of versatile networks of diseases, therapies, drug and genes with integrated knowledge of disease and therapy hierarchy, disease–gene and drug–therapy association and drug–target interactions. The study of these networks may bring new insights about the mechanism of disease perturbations and drug actions, and could aid in the discovery of new drug targets and novel drug design. VisANT accepts various inputs such as node/edge-lists, PSI-MI, GML. The resulting network can be saved, shared and published in different formats including VisANT XML format (VisML), edge-list and SVG. VisANT, along with the full user manual and tutorials, is available at http://visant.bu.edu.

## SUPPLEMENTARY DATA

Supplementary Data are available at NAR Online: Supplementary Instructions.

## FUNDING

Funding for open access charge: National Institutes of Health [R01GM103502-05].

*Conflict of interest statement.* None declared.
